# Microarray analysis and functional prediction of differentially expressed circular RNAs in acquired middle ear cholesteatoma

**DOI:** 10.1186/s12938-021-00960-x

**Published:** 2021-12-18

**Authors:** Shumin Xie, Li Jin, Tuanfang Yin, Jihao Ren, Wei Liu

**Affiliations:** 1 Department of Otolaryngology-Head and Neck Surgery, The Xiangya Hospital, Central South University, Hunan Provincial Key Lab, Otolaryngology Institute of Major Diseases, Changsha, 410008 Hunan China; 2 Department of Otolaryngology-Head and Neck Surgery, The Second Xiangya Hospital, Central South University, Changsha, 410011 Hunan China

**Keywords:** circRNA, Cholesteatoma, Etiopathogenesis, ceRNA, Microarray analysis

## Abstract

**Background:**

Middle ear cholesteatoma is characterized by hyper-proliferation of keratinocytes. Circular RNA (circRNA) plays an essential role in the pathogenesis of many proliferative diseases. However, the role of circRNA in the etiopathogenesis of middle ear cholesteatoma is rarely investigated so far. We aimed to investigate the differential expression profiling of circRNAs between acquired middle ear cholesteatoma and normal skin, and to identify potential circRNAs contributing to the etiopathogenesis of middle ear cholesteatoma. Microarray analysis and functional prediction were performed to investigate the circRNA expression profiling between middle ear cholesteatoma and normal skin. Validation of differentially expressed circRNAs was conducted by qRT-PCR. Prediction of m^6^A modification was also carried out.

**Results:**

Microarray analysis displayed that totally 93 up-regulated and 85 down-regulated circRNAs were identified in middle ear cholesteatoma. Through validation, expressions of hsa_circRNA_104327 and hsa_circRNA_404655 were significantly higher, while hsa_circRNA_000319 was significantly down-regulated in cholesteatoma. GO classification, KEGG pathway, and ceRNA network analyses suggested that these differentially expressed circRNAs might play important roles in the etiopathogenesis of middle ear cholesteatoma. Prediction of m^6^A modification exhibited that hsa_circRNA_000319 possessed 4 m^6^A sites with very high confidence, and hsa_circRNA_404655 had 3 m^6^A sites with high confidence.

**Conclusions:**

Our study revealed that these differentially expressed circRNAs might contribute to the etiopathogenesis of middle ear cholesteatoma. Further researches should be conducted to investigate the exact mechanism of these differentially expressed circRNAs in the etiopathogenesis of middle ear cholesteatoma. Targeting on these circRNAs may provide a new strategy for middle ear cholesteatoma therapy in the future.

**Supplementary Information:**

The online version contains supplementary material available at 10.1186/s12938-021-00960-x.

## Background

Middle ear cholesteatoma is a locally destructive squamous epithelial lesion with clinical features such as hyper-proliferation, migration, aggressiveness, and recurrence. Although middle ear cholesteatoma is pathologically benign, it progressively develops and leads to erosion of adjacent bony structures, resulting in vestibular dysfunction, labyrinthine fistulae, facial paralysis, hearing loss and serious intracranial complications [1, 2]. The incidence of middle ear cholesteatoma has been reported as high as 9.2 per 100,000 populations in Europe, while even higher in Asians [3]. Although a wide spectrum of hypotheses have been put forward, the exact etiopathogenesis of middle ear cholesteatoma is not clear yet. Since there is no effective drug for treating middle ear cholesteatoma, surgical resection is still the only option presently. However, surgical resection is often accompanied by dysfunction of middle and inner ear, which may cause further hearing loss [4]. Due to its recurrence, a few patients have to receive repeated surgeries. Meanwhile, for patients with poor general conditions, such as severe respiratory diseases, cardiovascular and cerebrovascular diseases, surgical resection cannot be performed.

Circular RNA (circRNA) is a special endogenous non-coding RNA with a unique circular structure, derived from exons or introns [5]. MicroRNA (miRNA) regulates the translation of message RNA (mRNA) at the post-transcriptional level via targeting complementary mRNAs and affecting several biological processes, such as cell differentiation, proliferation, and apoptosis [6]. Through binding to their targeted messenger RNA (mRNA) at the 3’-untranslated region, miRNA initiates degradation of mRNAs [7]. CircRNA is rich in miRNA response element (MRE), and functions as efficient miRNA sponges. Acting as a competing endogenous RNA (ceRNA), circRNA is able to suppress the activity of miRNA, release the inhibition of miRNA on its targeted mRNA, and promote the expression of target gene, through binding to miRNA [8]. CircRNA plays an important role in cell proliferation, differentiation, apoptosis, migration and body metabolism [9].

CircRNA participates in the pathogenesis of many proliferative diseases, such as hyper-proliferation of keratinocytes in psoriasis [10], hypoxia-induced proliferation of vascular endothelial cells [11], skeletal myoblast proliferation [12], and so on. Currently, hyper-proliferation of keratinocytes has been discovered in middle ear cholesteatoma, demonstrated by significantly high expression of numerous epithelial cell proliferation markers such as proliferating cell nuclear antigen (PCNA), Ki-67, cytokeratin (CK) 13, and CK16 in middle ear cholesteatoma epithelium [13–15]. However, the role of circRNA in the etiopathogenesis of middle ear cholesteatoma is rarely investigated so far.

In our research, we investigated the differential expression profiling of circRNAs between acquired middle ear cholesteatoma and normal skin by microarray technology. Expression of several selected circRNAs was validated through quantitative real-time polymerase chain reaction (qRT-PCR), purposing to recognize meaningful circRNAs contributing to the etiopathogenesis of middle ear cholesteatoma.

## Results

### Differential expressions of circRNAs in cholesteatoma and normal skin

**Fig. 1:**
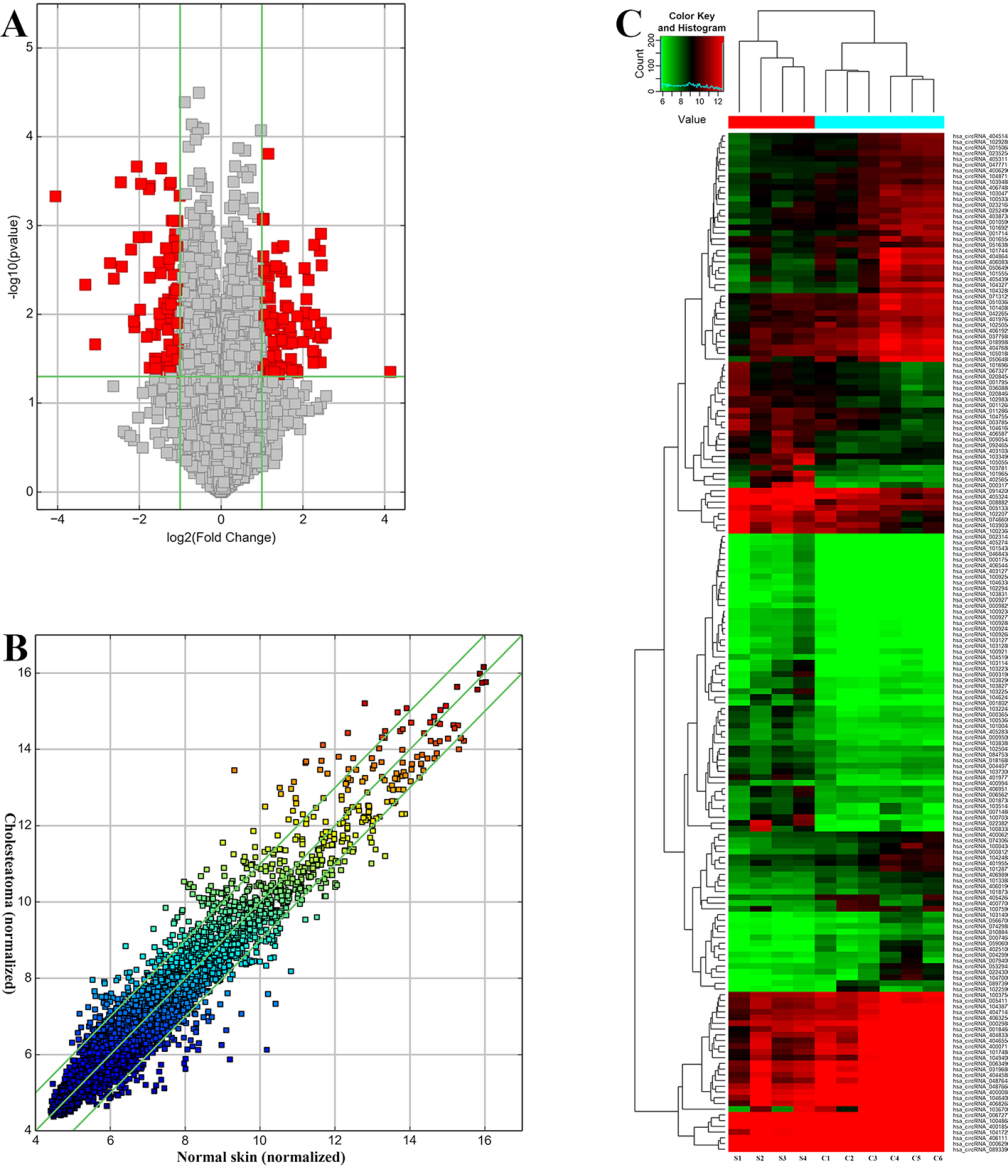
Microarray analysis of differentially expressed circRNAs in middle ear cholesteatoma and normal skin. **A** Volcano plot. **B** Scatter plot. **C** Hierarchical clustering analysis

In our study, a total of 13,562 expressed circRNAs were detected through microarray analysis. Compared with normal skin, totally 93 up-regulated (hsa_circRNA_104327, hsa_circRNA_404655, etc.) and 85 down-regulated (hsa_circRNA_000319, hsa_circRNA_048764, etc.) circRNAs were identified in middle ear cholesteatoma tissues with a value of fold change (FC) ≥ 2 (*P* < 0.05). The differentially expressed circRNAs were displayed in a volcano plot (Fig. 1A), and then subjected to a scatter plot (Fig. 1B) and hierarchical clustering analysis (Fig. 1C), to display the variation and differentiation between middle ear cholesteatoma and normal skin. Moreover, among these 178 significantly differentially expressed circRNAs, a total of 145 were exons. Discoveries in our study indicated that these differentially expressed circRNAs might contribute to the etiopathogenesis of middle ear cholesteatoma.

### Validation of differentially expressed circRNAs

**Fig. 2:**
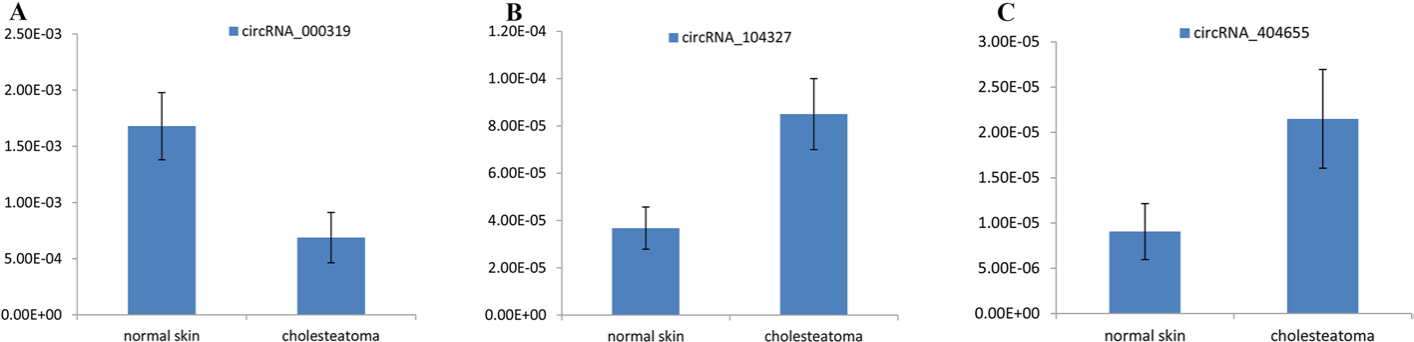
Validation of differentially expressed circRNAs through qRT-PCR. **A** Compared to normal skin, hsa_circRNA_000319 was significantly down-regulated (P < 0.05). **B** Compared to normal skin, hsa_circRNA_104327 was significantly up-regulated (P < 0.05). **C** Compared to normal skin, hsa_circRNA_404655 was significantly up-regulated (P < 0.05)

To validate the circRNAs microarray result, a total of 8 differentially expressed circRNAs were selected for qRT-PCR analysis. In detail, 5 up-regulated circRNAs (hsa_circRNA_103670, hsa_circRNA_048764, hsa_circRNA_404864, hsa_circRNA_104327, hsa_circRNA_404655), and 3 down-regulated circRNAs (hsa_circRNA_101965, hsa_circRNA_000319, hsa_circRNA_100927) were validated. The validation result was in accordance with the microarray discoveries partly: compared with normal skin, expressions of hsa_circRNA_104327 and hsa_circRNA_404655 were significantly higher in middle ear cholesteatoma (*P* < 0.05), while the expression of hsa_circRNA_000319 significantly decreased (*P* < 0.05) (Fig. 2).

### Gene ontology (GO) and Kyoto Encyclopedia Genes and Genomes (KEGG)

**Fig. 3:**
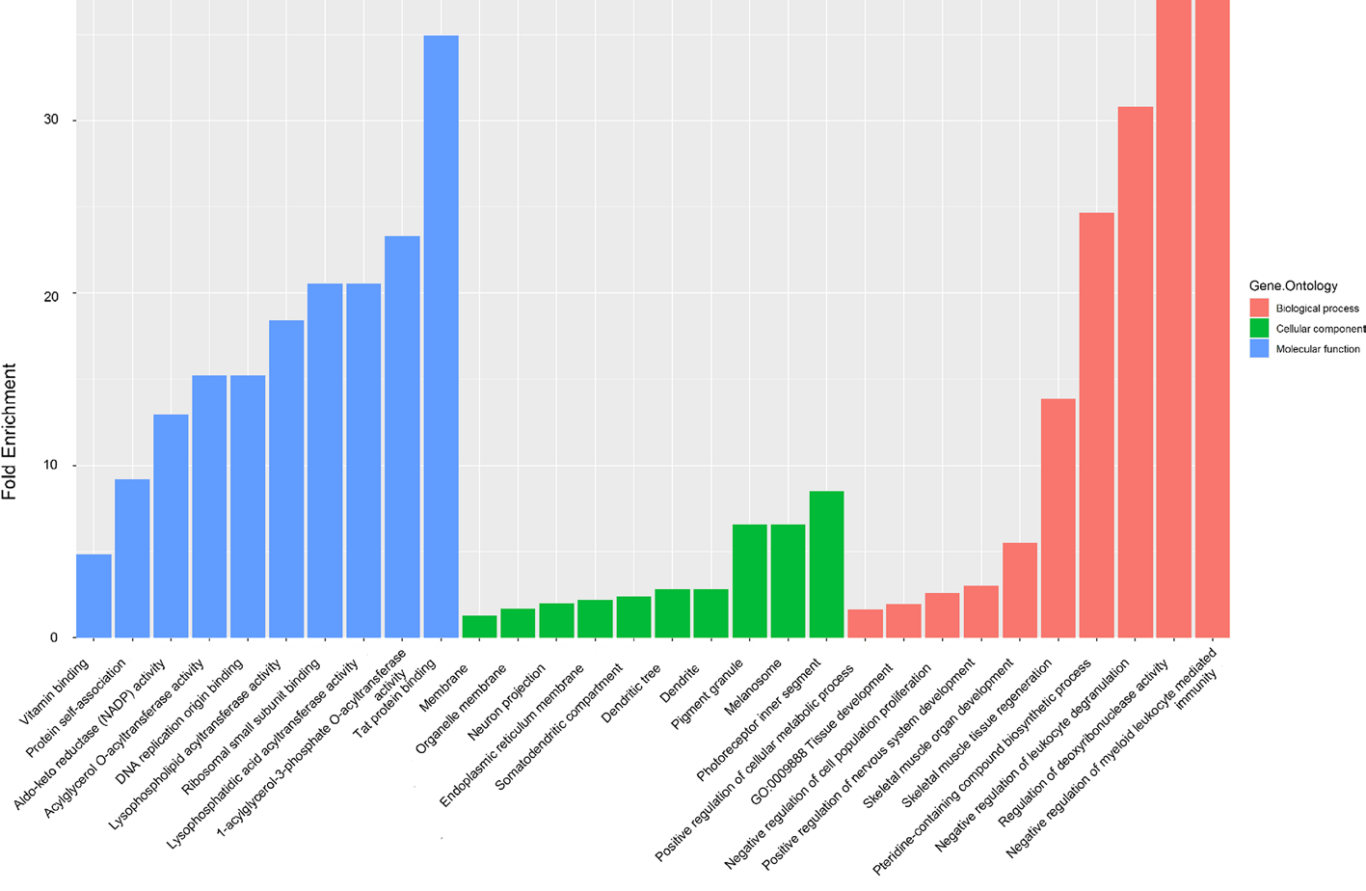
Go classification of validated differentially expressed circRNAs

**Fig. 4:**
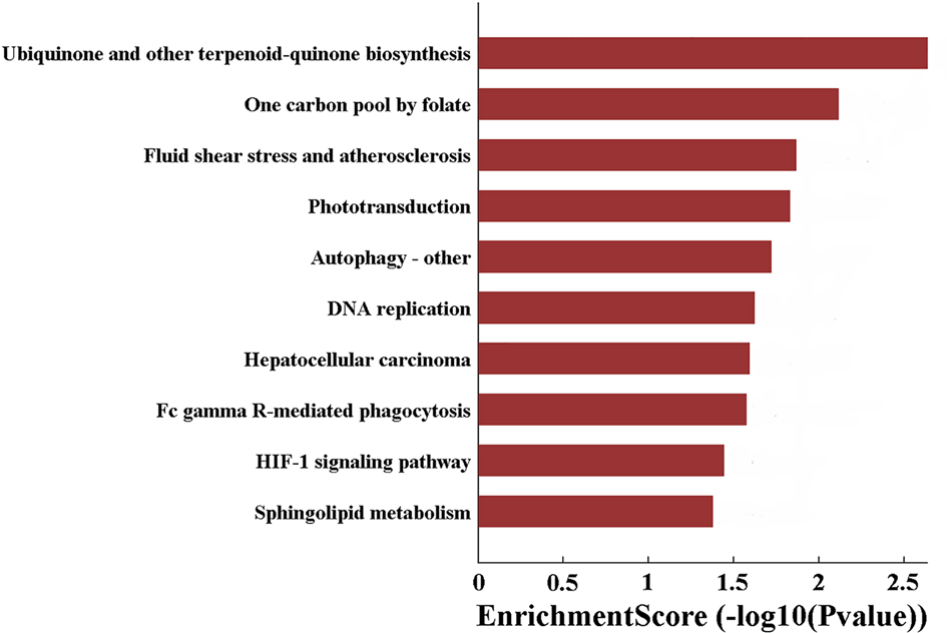
KEGG pathway analysis of validated differentially expressed circRNAs

To preliminarily investigate the function of differentially expressed circRNAs in middle ear cholesteatoma, GO classification and KEGG pathway analysis were carried out on presumptive target genes of validated circRNAs (hsa_circRNA_104327, hsa_circRNA_404655, and hsa_circRNA_000319). Three domains including biological process (BP), cellular component **(**CC) and molecular function (MF) in GO classification were investigated. In this research, a total of 205 BP, 28 CC, and 28 MF domains enriched significantly (*P* < 0.05). The top-10 enriched BP, CC, and MF domains in GO classification are displayed in Fig. 3. Meanwhile, as for KEGG pathway analysis, a total of 11 signaling pathways enriched significantly (Fig. 4).

### ceRNA network analysis

Based on the ceRNA hypothesis, circRNAs can bind to miRNAs and suppress the activity of miRNAs through MRE. In our study, 3 validated differentially expressed circRNAs with statistical significance including hsa_circRNA_000319, hsa_circRNA_104327, and hsa_circRNA_404655 were selected to perform a circRNA–miRNA–mRNA ceRNA network analysis.

**Fig. 5:**
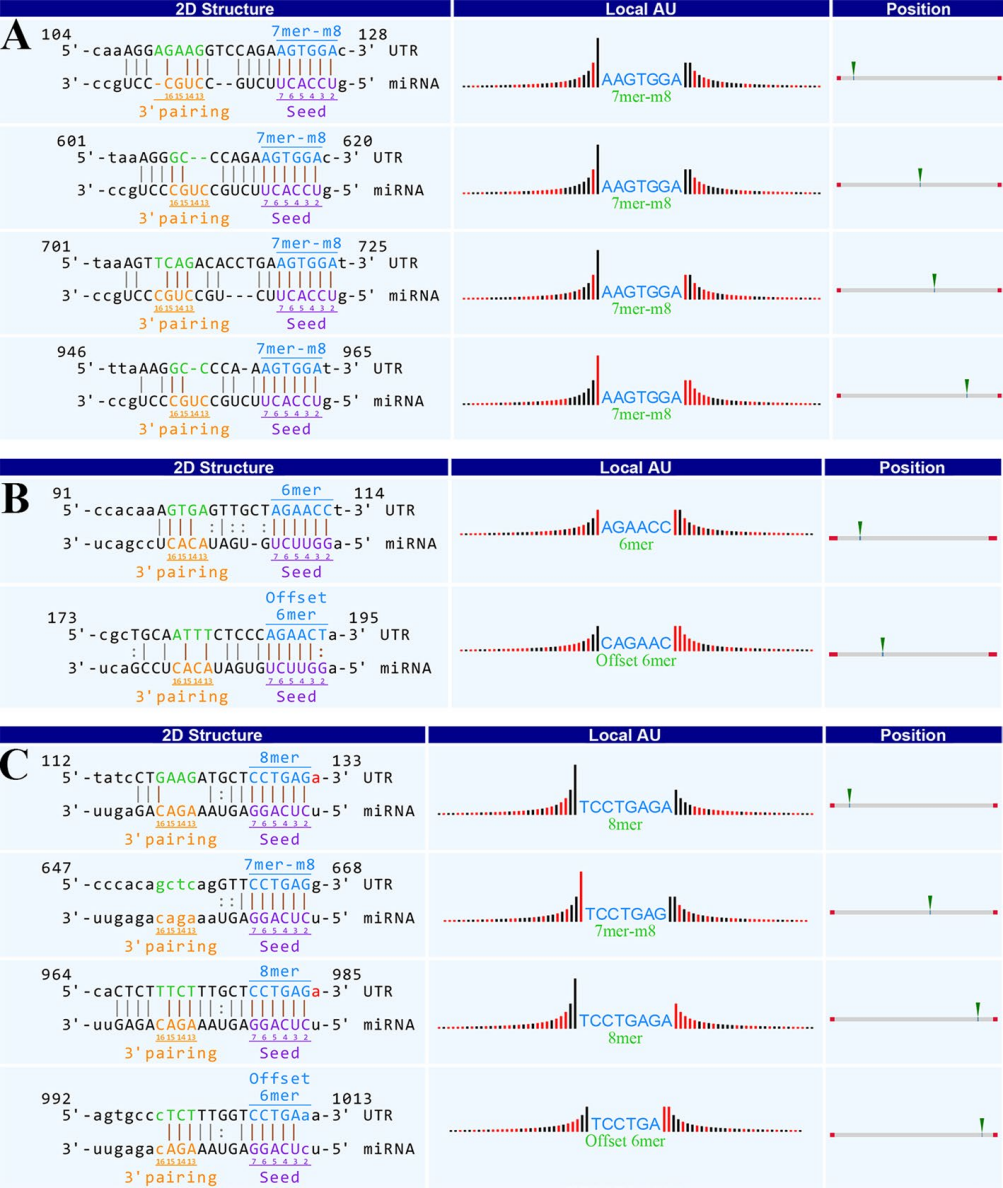
Interactions between validated differentially expressed circRNAs. **A** Interactions between hsa_circRNA_000319 and hsa-miRNA-4436b-5p. **B** Interactions between hsa_circRNA_104327 and miRNA-152-5p. **C** Interactions between hsa_circRNA_404655 and miRNA-3664-3p

The ceRNA network was composed of 3 circRNAs, 309 miRNAs, and 131 mRNAs (Additional file 1: Fig. S1). We discovered that hsa_circRNA_000319 interacted with hsa-miRNA-4436b-5p, hsa_circRNA_104327 interacted with miRNA-152-5p, and hsa_circRNA_404655 interacted with miRNA-3664-3p, with a complementarity based on 7mer-m8, 6mer, offset 6mer, or 8mer sites (Fig. 5). Moreover, in our previous study [16], compared with normal skin, the expression of miRNA-4436b-5p was demonstrated to be significantly up-regulated in middle ear cholesteatoma (FC > 2, *P* < 0.05), while miRNA-152-5p and miRNA-3664-3p were obviously down-regulated (FC < 0.5, *P* < 0.05), which were synchronous with expressions of hsa_circRNA_000319, hsa_circRNA_104327, and hsa_circRNA_404655 in middle ear cholesteatoma. Additionally, several targeted mRNAs for miRNA-3664-3p were discovered, including IQ motif-containing GTPase activating protein 3 (IQGAP3), receptor tyrosine kinase-like orphan receptor 2 (ROR2), ephrin type-B receptor 1 (EPHB1), nucleophosmin 1 (NPM1), chromosome condensation 2 (RCC2), nuclear receptor coactivator 5 (NCOA5), and so on.

### Prediction of N^6^-methyladenosine (m^6^A) on differentially expressed circRNAs

**Fig. 6:**
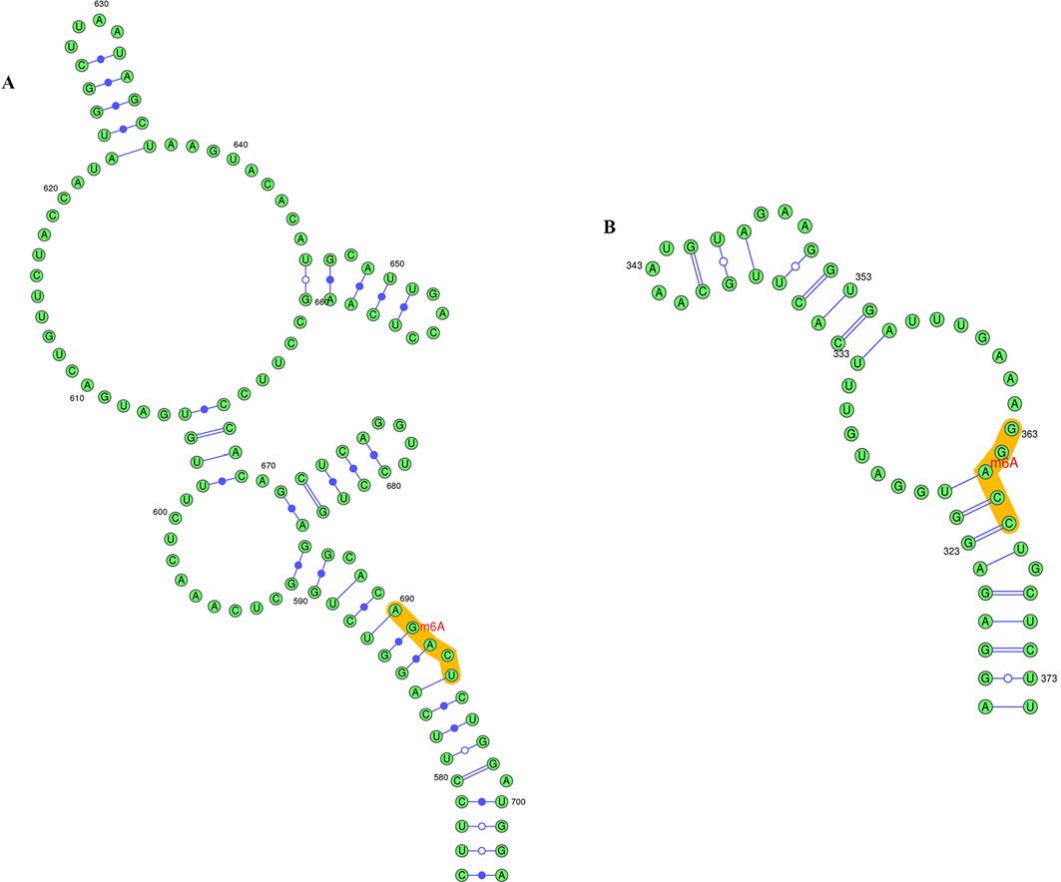
**A** local structure of hsa_circRNA_404655 with m^6^A modification on the 692nd base position. **B** local structure of hsa_circRNA_000319 with m^6^A modification on the 365th base position

m^6^A is a post-transcriptional methylation modification that widely presents at the adenosine bases of RNA transcripts. This modification has been suggested to regulate the function of circRNAs. In our study, we discovered that hsa_circRNA_000319 possessed 4 m^6^A sites with very high confidence in total, and hsa_circRNA_404655 had 3 m^6^A sites with high confidence, while no m^6^A site with very high or high confidence was found for hsa_circRNA_104327. Details are displayed in Table 1 and Fig. 6.

**Table 1:** Prediction of m^6^A on differentially expressed circRNAs

circRNAs	Position	Sequence context	Combined score	Decision of m^6^A site
hsa_circRNA_000319	365	UGAAA GGACC UGCUC	0.845	Very high confidence
hsa_circRNA_000319	418	GAUAU UGACA UUCAU	0.831	Very high confidence
hsa_circRNA_000319	511	GAAAU UGACU UGAAU	0.817	Very high confidence
hsa_circRNA_000319	1147	AGUAU GAACA UUGAG	0.730	Very high confidence
hsa_circRNA_404655	692	GGCAC AGACU CUGGA	0.668	High confidence
hsa_circRNA_404655	797	AAGUU GAACU GCAAU	0.619	High confidence
hsa_circRNA_404655	1068	ACUAC AGACU UAGUG	0.618	High confidence

*circRNA* circular RNA

## Discussion

CircRNA is a special endogenous non-coding RNA with a unique ring structure, which regulates gene expression at the post-transcriptional level and plays an important role in a series of life processes, such as cell proliferation, differentiation, apoptosis, migration and metabolism [5, 9]. However, there are few studies on circRNAs in middle ear cholesteatoma, and the role of circRNAs in the etiopathogenesis of middle ear cholesteatoma is rarely investigated so far. We totally identified 93 significantly up-regulated and 85 down-regulated circRNAs in cholesteatoma compared with normal skin. Moreover, qRT-PCR was performed to verify the microarray discoveries by detecting the expression of 8 selected circRNAs. Expressions of hsa_circRNA_104327, hsa_circRNA_404655 and hsa_circRNA_000319 were in line with the microarray results, suggesting that these three circRNAs might play a pivotal role in the etiopathogenesis of middle ear cholesteatoma. The difference between microarray results and qRT-PCR validation might be caused by the false-positive results from microarray analysis. Furthermore, a validation with a large sample size will be conducted in our further research.

CircRNA is closely related to the pathogenesis of a variety of proliferative diseases, such as psoriasis [10], hypoxia-induced vascular endothelial cell proliferation [11] and so on. In the process of these proliferative diseases, circRNA regulates the expression of related target genes as miRNA "sponge" and participates in the etiopathogenesis of these diseases. Wang et al. [12] found that circTitin activated insulin-like growth factor 2 (IGF2)/phosphatidylinositol 3-kinase (PI3K)/Akt signal pathway through adsorption of miRNA-432, which led to the proliferation and differentiation of skeletal myoblasts. In addition, circRNA is also closely related to tumorigenesis. Studies discovered an abnormal expression of circRNAs in many kinds of tumor tissues, including breast cancer [17], liver cancer [18] and esophageal cancer [19]. Additionally, circRNAs were demonstrated to participate in tumor cell hyper-proliferation, invasion and metastasis. For example, the expression of circSMARCA5 was significantly down-regulated in hepatocellular carcinoma, and was closely related to the hyper-proliferation, invasion and metastasis of hepatocellular carcinoma cells. Transfection of circSMARCA5 into the hepatocellular carcinoma cells up-regulated the expression of tissue inhibitor of matrix metalloproteinase 3 (TIMP3) through adsorption of miRNA-17-3p and miRNA-181b-5p, and ultimately inhibited the proliferation, invasion and metastasis of hepatocellular carcinoma cells [18]. A study found that hsa_circRNA_102747 was significantly down-regulated in cholesteatoma and acted as the endogenous sponge of miRNA-21-3p [20], a miRNA promoting excessive proliferation of cholesteatoma keratinocytes [21]. Meanwhile, hsa_circRNA_101458 was also obviously down-regulated in cholesteatoma and was able to interact with miRNA-let-7a-3p [20], a miRNA which was confirmed to suppress proliferation of keratinocytes in cholesteatoma [22]. Consequently, circRNAs might play an important role in the etiopathogenesis of middle ear cholesteatoma.

So far, the exact function of circRNAs in middle ear cholesteatoma still remains unclear. According to this research and our previous research [16], expression of hsa_circRNA_404655 significantly increased in middle ear cholesteatoma, while miRNA-3664-3p obviously decreased and interacted with hsa_circRNA_404655. The decreased expression of miRNA-3664-3p might lead to an increased expression of growth differentiation factor 15 (GDF15), which consequently increased the expression of p53 and promoted tumor progression in spinal cord glioblastoma [23]. Although cholesteatoma is pathologically benign, it is locally invasive and can lead to bone resorption. We speculate that hsa_circRNA_404655, as a sponge of miRNA-3664-3p, may participate in the proliferation and local invasion of cholesteatoma. Moreover, we also discovered several targeted mRNAs for hsa_circRNA_404655 and miRNA-3664-3p, including IQGAP3, ROR2, EPHB1, NPM1, RCC2, NCOA5, and so on. Although these targeted mRNAs have not been investigated in middle ear cholesteatoma, their functions were explored in other proliferative and invasive diseases. For instance, IQGAP3, EPHB1, ROR2 and RCC2 were demonstrated to obviously promote cell migration, invasion, and epithelial-to-mesenchymal transition in hepatocellular carcinoma, medulloblastoma and breast carcinoma, respectively [24–27]. Moreover, NPM1 contributed to cell proliferation through phosphatase and tensin homologue deleted on chromosome ten (PTEN) inactivation [28], while NCOA5 promoted proliferation, migration and invasion of colorectal cancer cells via activation of PI3K/Akt pathway [29]. Similarly, epithelial-to-mesenchymal transition, PTEN inactivation and PI3K/Akt pathway activation were also noticed in middle ear cholesteatoma [30, 31]. These results provide new insights into the pathogenesis of cholesteatoma and suggest that circRNAs may be a potential target for the treatment of cholesteatoma. The exact mechanism needs to be further investigated.

Through KEGG pathway analysis, autophagy and hypoxia inducible factor-1 (HIF-1) signaling pathway were identified. Li et al. [32] proposed that enhanced autophagy might contribute to the pathogenesis of middle ear cholesteatoma through PI3K/Akt pathway activation. On the contrary, Ho et al. [33] put forward that autophagy was significantly suppressed in cholesteatoma. Moreover, activated HIF-1 pathway was detected in middle ear cholesteatoma, indicating that cholesteatoma might be hypoxic [34]. Furthermore, KEGG pathway analysis exhibited several potential signaling pathways, which broadened our horizons on the etiopathogenesis of middle ear cholesteatoma.

More and more evidence shows that m^6^A modification can regulate the function of circRNAs. For instance, m^6^A modification was able to initiate circRNAs translation through the cooperation of eukaryotic translation initiation factor 4 gamma 2 (eIF4G2) and m^6^A reader YTH domain family 3 (YTHDF3) [35]. Park et al. [36]. demonstrated that m^6^A modification could reduce the stability of circRNAs. In addition, m^6^A modification can also regulate cytoplasmic output. In previous research, YTH domain-containing protein 1 (YTHDC1) mediated the nuclear output of circNSUN2 in a m^6^A methylation-dependent manner, thereby increasing cytoplasmic expression of circNSUN2 and promoting metastasis of colorectal cancer [37]. Moreover, it has been reported that foreign circRNAs were able to induce T cell activation, antibody production, and anti-tumor immunity, and m^6^A modification on circRNAs inhibited immune gene activation [38]. In our study, we predicted that hsa_circRNA_000319 possessed 4 m^6^A sites with very high confidence in total, and hsa_circRNA_404655 had 3 m^6^A sites with high confidence. Experiments to verify m^6^A modification on differentially expressed circRNAs will be performed in our further study.

## Conclusion

In summary, 93 up-regulated and 85 down-regulated circRNAs were identified in middle ear cholesteatoma tissues in the current study. Compared with normal skin, the qRT-PCR validation for differentially expressed circRNAs demonstrated that hsa_circRNA_104327 and hsa_circRNA_404655 were expressed significantly higher while hsa_circRNA_000319 expression significantly decreased in cholesteatoma tissue, suggesting that circRNAs may contribute to the pathogenesis of middle ear cholesteatoma. Further researches should be conducted to investigate the exact mechanism of these differentially expressed circRNAs in the pathogenesis of middle ear cholesteatoma. Targeting on theses circRNAs may provide a new strategy for middle ear cholesteatoma therapy in the future.

## Materials and methods

### Patients and samples

A total of 16 acquired middle ear cholesteatoma tissues were obtained from patients undergoing cholesteatoma surgery between August 2019 and December 2019. Meanwhile, 14 normal skin (from incision ethier endaural or postaturicular) samples were collected to be recognized as controls. All samples were stored in liquid nitrogen at once for microarray analysis and qRT-PCR validation.

### RNA extraction

Total RNAs were extracted from 6 middle ear cholesteatoma and 4 normal skin tissues using TRIzol reagent (Invitrogen; USA) according to the manufacturer's instructions. Then, RNasey mini kit (QIAGEN, German) was used to purify RNAs. Meanwhile, RNA concentrations were determined through NanoDrop ND-1000 instrument (Thermo, USA), and RNA integrity was assessed through gel electrophoresis.

### CircRNA labeling and microarray hybridization

The sample preparation and circRNA microarray hybridization were performed based on the Arraystar’s standard protocols. Briefly, total RNAs were digested with Rnase R (Epicentre, Inc.) to remove linear RNAs and enrich circRNAs. Then, the enriched circRNAs were amplified and transcribed into fluorescent cRNA utilizing a random priming method (Arraystar Super RNA Labeling Kit; Arraystar). The labeled cRNAs were hybridized onto the Arraystar Human circRNA Array V2 (8 × 15K, Arraystar), and incubated for 17 h at 65 °C in an Agilent Hybridization Oven. After having washed the slides, the arrays were scanned by the Agilent Scanner G2505C.

### Image acquisition and microarray analysis of circRNAs

Agilent Feature Extraction software (version 11.0.1.1) was used to analyze acquired array images. Quantile normalization and subsequent data processing were performed using the R software limma package. Differentially expressed circRNAs with statistical significance between middle ear cholesteatoma and normal skin were identified using FC cutoff or volcano plot filtering, respectively. The value of FC ≥ 2 and *P* < 0.05 was regarded as significantly differentially expressed. Hierarchical Clustering was performed to show the distinguishable circRNAs expression pattern between middle ear cholesteatoma and normal skin samples. Finally, the circRNA–miRNA interaction was predicted with Arraystar's home-made miRNA target prediction software based on TargetScan (http://www.targetscan.org) and miRanda (http://www.microrna.org).

### Validation for differentially expressed circRNAs by qRT-PCR

Total cellular RNA was, respectively, purified from 10 cholesteatoma and 10 normal skin tissues using Trizol reagent (Invitrogen, USA), precipitated with alcohol, and then stored at − 70 °C until use. Extracted RNA was quantified by measuring optical density at 260 nm, and then 0.4 µg RNA was reversely transcribed to cDNA in a 20-µl reaction system according to the manufacturer’s protocol. Then, quantitative PCR reactions were conducted using QuantStudioTM 5 System (Applied Biosystems, USA) with 2 µl cDNA sample adding into a 8-µl reaction system. Amplification conditions were controlled as follows: 95 °C for 10 min for 1 cycle, followed by 40 cycles of 95 °C for 10 s and 60 °C for 60 s. The specific amplification primer pairs are shown in Table 2.

**Table 2:** Primers for RT-qPCR validation

Gene name	Primer sequences
β-actin	F:5' GTGGCCGAGGACTTTGATTG3' R:5’ CCTGTAACAACGCATCTCATATT3’
hsa_circRNA_103670	F:5’ ATAACAATCTGTTACGGGTTTT 3’ R:5’ GGCATCCCTATTAGTCTTTCAA 3’
hsa_circRNA_048764	F:5’ TGCCCTCTCCCTGAAATAAAG 3’ R:5’ GCAGTGCGGGAAACTTCTGT 3’
hsa_circRNA_404864	F:5’ TCTCCCAAGGAAGATACCGA 3’ R:5’ GCTCAGAGCAGTGCACATTATT 3’
hsa_circRNA_104327	F:5’ TGTGTCAAATTTGTTCAAGACA 3’ R:5’ CAGGTTCTAGCAACTCACTTTG 3’
hsa_circRNA_404655	F:5’ CAGCACTCCACAGCATCCACTA 3’ R:5’ TCCATTTCAATGGTAGCCTGCA 3’
hsa_circRNA_101965	F:5’ GTCTTCCAGATCCACCAGGTT 3’ R:5’ TGCCATCTGTCAGAAACTTGAT 3’
hsa_circRNA_100927	F:5’ GATAAAAGTGGATTGCAAGACT 3’ R:5’ TTCAAATAAACTGTCTGCCAAC 3’
hsa_circRNA_000319	F:5’ TGTAGCTGTGGATCTACCAAAA 3’ R:5’ ACTTTGGGGCCTTTCAAGC 3’

*circRNA* circular RNA, *F* forward, *R* reverse

### ceRNA network analysis

Significantly differentially expressed circRNAs including hsa_circRNA_000319, hsa_circRNA_104327, and hsa_circRNA_404655 were selected to perform ceRNA network analysis. The cytoscape software V3.5.0 (San Diego, CA, USA) was adopted to construct circRNA-associated ceRNA network.

### GO and KEGG pathway analysis

GO classification (http://www.geneontology.org) and KEGG pathway analysis (http://www.genome.jp/kegg/) were applied to explore functions of target genes in the pathogenesis of middle ear cholesteatoma. The GO classification covers three domains including BP, CC, and MF, and describes the gene functions. Pathway analysis was conducted to predict molecular interactions and reaction networks through mapping genes to KEGG pathways. A *P* < 0.05 was considered to be statistically significant.

### Prediction of m^6^A modification on differentially expressed circRNAs

Sequence-based RNA adenosine methylation site predictor (SRAMP) website (http://www.cuilab.cn/sramp) was searched in order to explore the m^6^A modification on differentially expressed circRNAs. Significantly differentially expressed circRNAs including hsa_circRNA_000319, hsa_circRNA_104327, and hsa_circRNA_404655 were selected to conduct the prediction of m6A modification sites on the RNA sequences of these 3 circRNAs.

## Supplementary Information

**Additional file 1: Figure S1.** The ceRNA network of validated differentially expressed circRNAs.

## Data Availability

All data generated and analyzed during this study are included in this published article, detailed data have been uploaded in https://www.ncbi.nlm.nih.gov/geo/query/acc.cgi?acc=GSE171006.

## Abbreviations

circRNA: Circular RNA
miRNA: MicroRNA
mRNA: Message RNA
MRE: MiRNA response element
ceRNA: Competing endogenous RNA
PCNA: Proliferating cell nuclear antigen
CK: Cytokeratin
qRT-PCR: Quantitative real-time polymerase chain reaction
GO: Gene Ontology
KEGG: Kyoto Encyclopedia Genes and Genomes
BP: Biological process
CC: Cellular component
MF: Molecular function
m^6^A: N6-methyladenosine
IQGAP3: IQ motif-containing GTPase activating protein 3
ROR2: Receptor tyrosine kinase-like orphan receptor 2
EPHB1: Ephrin type-B receptor 1
NPM1: Nucleophosmin 1
RCC2: Chromosome condensation 2
NCOA5: Nuclear receptor coactivator 5
IGF2: Insulin-like growth factor 2
PI3K: Phosphatidylinositol 3-kinase
TIMP3: Tissue inhibitor of matrix metalloproteinase 3
GDF15: Growth differentiation factor 15
PTEN: Phosphatase and tensin homologue deleted on chromosome ten
HIF-1: Hypoxia inducible factor-1
eIF4G2: Eukaryotic translation initiation factor 4 gamma 2
YTHDF3: YTH domain family 3
YTHDC1: YTH domain-containing protein 1
RIG-I: Retinoic acid-inducible gene I
