# Supplementary figures and images for: Microarray analysis and functional prediction of differentially expressed circular RNAs in acquired middle ear cholesteatoma
Source: Biomed Eng Online. 2021 Dec 18;20:129. doi: 10.1186/s12938-021-00960-x (PMC8684697; doi:10.1186/s12938-021-00960-x)

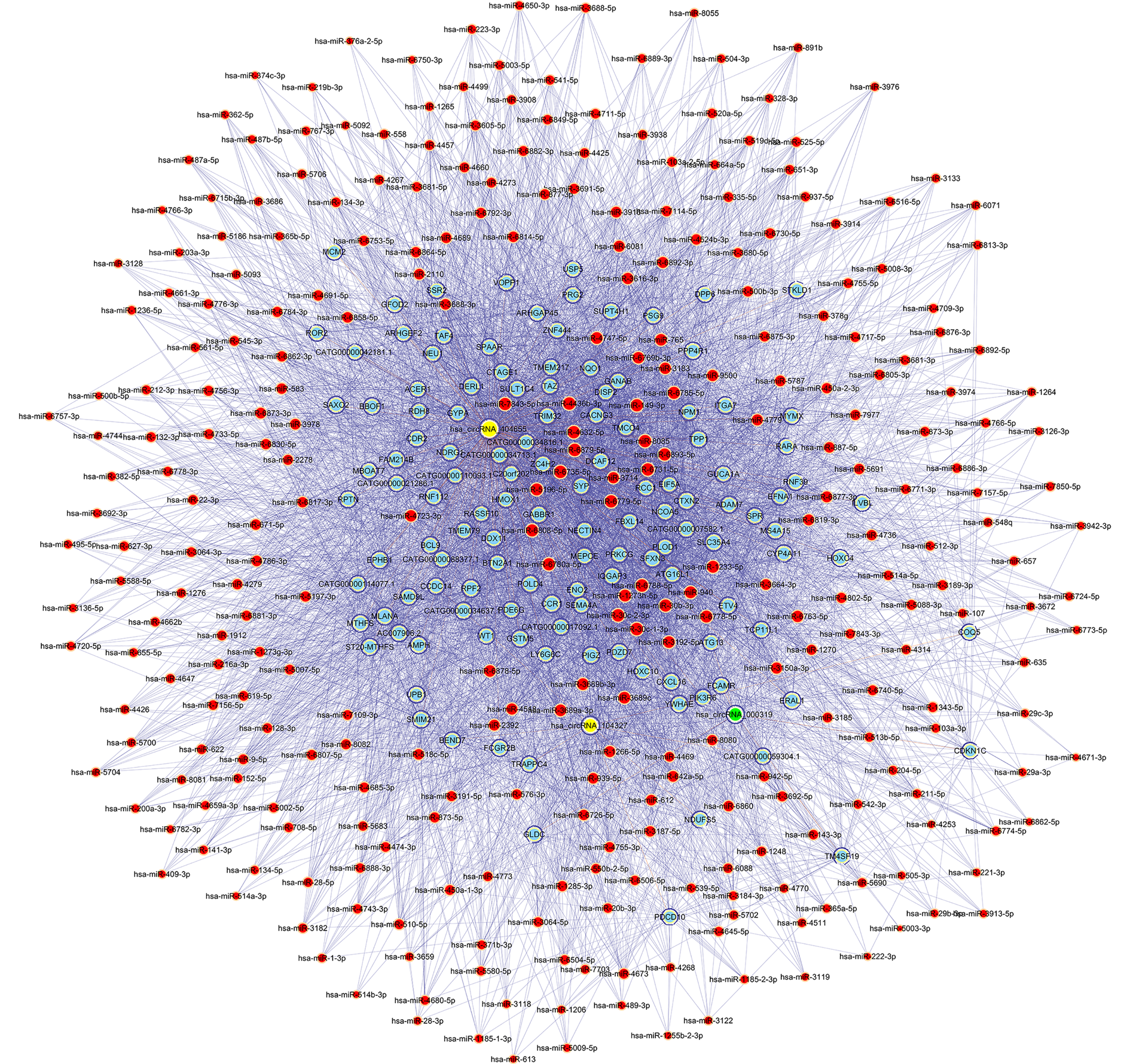

Supplement: Supplementary file 1 — Additional file 1: Figure S1. The ceRNA network of validated differentially expressed circRNAs. [file 12938_2021_960_MOESM1_ESM.tif]
